# Lovastatin Targets the USP14–Survivin Axis to Suppress Triple-Negative Breast Cancer via Ubiquitin-Mediated Proteasomal Degradation

**DOI:** 10.3390/cells14110816

**Published:** 2025-05-31

**Authors:** Li Zhou, Chanjuan Zheng, Siyu Ding, Zhiyu Wang, Yiyuan Yang, Yian Wang, Guangchun He, Shujun Fu, Xiyun Deng

**Affiliations:** 1 Key Laboratory of Translational Cancer Stem Cell Research, Department of Pathophysiology, Hunan Normal University, Changsha 410013, China; zhouli_723@163.com (L.Z.); zhengchanjuan@hunnu.edu.cn (C.Z.); dingsiyu@hunnu.edu.cn (S.D.); wzy1222@hunnu.edu.cn (Z.W.); yiyuanyang6@126.com (Y.Y.); wangyian@hunnu.edu.cn (Y.W.); emilygch2001@yahoo.com (G.H.); shujunfu2020@hunnu.edu.cn (S.F.); 2 Department of Pathology, National Clinical Research Center for Geriatric Disorders, Xiangya Hospital, Central South University, Changsha 410008, China

**Keywords:** TNBC, Survivin, lovastatin, deubiquitination, USP14

## Abstract

Triple-negative breast cancer (TNBC), characterized by the absence of estrogen receptor (ER), progesterone receptor (PR), and human epidermal growth factor receptor type 2 (HER2) expression, represents a therapeutic challenge due to its aggressive nature and limited treatment options. Here, we identified the cholesterol-lowering drug lovastatin (LV) as a potent apoptosis-inducing agent in TNBC. Mechanistically, LV disrupts the interaction between the deubiquitinating enzyme USP14 and Survivin, a key anti-apoptotic protein, enhancing polyubiquitination and the proteasomal degradation of Survivin. The overexpression of USP14 was found to stabilize Survivin and rescue LV-induced apoptosis and tumor suppression in vitro and in vivo, whereas USP14 silencing or inhibition with IU1 (a USP14-specific inhibitor) enhanced Survivin turnover and synergized with LV to suppress colony formation in TNBC cells. Clinical relevance was demonstrated through bioinformatic analysis and immunohistochemistry, revealing that elevated Survivin expression in TNBC tissues correlated with poor prognosis and is significantly upregulated in TNBC versus non-TNBC tissues. Our findings identify the USP14–Survivin axis as a potential therapeutic target and highlight LV as a promising candidate for TNBC treatment.

## 1. Introduction

Breast cancer (BC) remains the leading cause of cancer-related deaths among women globally, with its incidence continuing to rise each year [1,2]. Triple-negative breast cancer (TNBC) is a specific type of BC defined by the lack of expression of the estrogen receptor (ER), the progesterone receptor (PR), and human epidermal growth factor receptor type 2 (HER2), accounting for approximately 10–20% of all BC cases. TNBC is particularly noted for its high malignancy and aggressiveness, contributing to its challenging treatment landscape [3,4]. In recent years, novel therapeutic options approved by the FDA, such as PARP inhibition, immune checkpoint-based immunotherapy, and antibody-drug conjugates, have shown promise in subsets of TNBC patients [5]. Even so, the prognosis of the majority of TNBC patients is still poor due to the high rate of drug resistance and recurrence [6,7]. Therefore, in-depth investigation into the mechanism behind the aggressive behavior of TNBC and an exploration of effective treatment strategies are of great clinical significance to improve the prognosis of patients.

Survivin, also known as apoptosis inhibitor 4, which belongs to the inhibitor of apoptosis protein (IAP) family, is essential for cell division and survival through its ability to suppress cell death. As the most potent IAP family member found to date [8,9], Survivin has been demonstrated to be highly expressed in almost all human malignancies, including breast [10], gastric [11], lung [12], liver [13], and ovarian cancers [14]. In addition, previous reports have also identified Survivin as a biomarker and potential prognostic indicator for BC [15,16]. In TNBC, the overexpression of Survivin has been closely linked to apoptosis resistance and is associated with clinicopathological factors and survival outcomes [17,18,19]. Therefore, targeting Survivin represents a promising therapeutic strategy for the treatment of TNBC.

LV is an active ingredient in red yeast rice and is rich in plants such as oyster mushrooms [20,21]. In addition to its lipid-lowering effects, LV possesses anti-bacterial, anti-inflammatory, neuroprotective, and anti-cancer properties [22,23]. In recent years, the anti-cancer properties of LV have attracted widespread attention. By blocking the mevalonate pathway, it disrupts the post-translational modification of small GTP-binding proteins like Ras, impairing tumor cell proliferation, migration, and invasion. Additionally, it induces apoptosis and inhibits angiogenesis in cancer cells. We have previously demonstrated that LV exhibits pro-apoptotic activities in TNBC cells and in a xenograft tumor model [24]. However, the mechanism by which LV induces apoptosis and its translational significance in TNBC remain largely elusive.

In this study, we investigated the mechanism underlying the apoptosis-inducing effects of LV in TNBC. We found that LV induces apoptosis through the downregulation of Survivin in TNBC cells. USP14 is a proteasome-associated deubiquitinating enzyme, which is activated hundreds of times by the proteasome [25,26]. Mechanistically, LV disrupts the interaction between Survivin and the deubiquitinase USP14, resulting in enhanced ubiquitination and the proteasomal degradation of Survivin. Furthermore, a higher expression of Survivin is correlated with a poorer prognosis in TNBC patients. Our findings identify the USP14–Survivin axis as a potential therapeutic target for TNBC.

## 2. Materials and Methods

### 2.1. Cell Lines and Cell Culture

TNBC cell lines MDA-MB-231, BT549, and HCC1806 were obtained from the CAS Shanghai Cell Resources Center (Shanghai, China). MDA-MB-231 cells were cultured in DMEM/F12 medium (Gibco, Grand Island, NY, USA), while BT549 and HCC1806 cells were maintained in RPMI 1640 medium (Gibco, Grand Island, NY, USA) supplemented with 10% (*v*/*v*) fetal bovine serum (FBS) and 1% penicillin–streptomycin solution (P/S). All cultures were incubated at 37 °C in a 5% CO_2_ incubator with controlled humidity, following the standard cell culture procedure.

### 2.2. Reagents and Antibodies

Lovastatin (LV, ab120614) was purchased from Abcam (Cambridge, UK), dissolved in DMSO to 100 mM, and stored at −80 °C before use. Antibodies against cleaved-Caspase 3 (IB: 1:1000; IF: 1:400; IHC: 1:2000; #9661), cytochrome C (1:1000; #11940), α-tubulin (1:5000; #2125), Bax (1:1000; #14796), VDAC1 (1:3000; #4866), Survivin (IB: 1:1000; IHC: 1:1000; #2808), p-H3 Ser10 (IHC: 1:1000; IF: 1:800; #3377), and ubiquitin (IB: 1:1000; #3936) were sourced from Cell Signaling Technology (Danvers, MA, USA). Anti-Ki67 antibodies (IB: 1:1000; IHC: 1:200; ab16667) were obtained from Abcam (Cambridge, UK). Anti-β-actin (1:5000; #A5316) and anti-Flag (1:10,000, F1804) antibodies were purchased from Sigma-Aldrich (St. Louis, MO, USA). The antibodies against USP14 (IB: 1:1000; IHC: 1:500; PA5-30300) were purchased from Thermo Fisher Scientific (Waltham, MA, USA). The inhibitors used in this study, including Necrostatin-1 (Nec-1) (S8037), z-VAD-FMK (S7023), 3-MA (S2767), MG132 (S2619), IU1 (S7134), and cycloheximide (CHX) (S7418), were purchased from Selleck Chemicals (Houston, TX, USA).

### 2.3. Plasmid Constructs and Lentiviral Infection

Lentiviral plasmids containing sh*Survivin*#1, sh*Survivin*#2, sh*USP14*#1, and sh*USP14*#2 were obtained from Thermo Fisher Scientific (Waltham, MA, USA). The lentiviral packaging plasmid *psPAX2* and the envelope plasmid *pMD2.G*, along with the control *shNC* plasmid, were acquired from Addgene (Cambridge, MA, USA). A total of 20 μL Lipofectamine 2000/250 μL OPTI-MEM was mixed with DNA (4 μg target + 3 μg *psPAX2* + 1 μg *pMD2.G* in 250 μL OPTI-MEM), incubated for 20 min, and added to cells. The medium was replaced after overnight incubation. Supernatants were collected at 48 h and 72 h, and were stored at −80 °C. The expression construct *pCMV6-Entry-USP14* was sourced from OriGene (Rockville, MD, USA). Stable knockdown cell lines were generated by lentiviral transduction following a standard protocol: the cells were infected with lentivirus at an MOI (Multiplicity of Infection) in the presence of 1‰ polybrene (Merck Millipore, Billerica, MA, USA), and stable clones were selected with 2 μg/mL puromycin (Merck Millipore, Billerica, MA, USA) at 72 h post-transduction. Stable knockdown cell lines for *Survivin* were generated in MDA-MB-231, BT549, and HCC1806 cells, while *USP14* knockdown was established in MDA-MB-231 and BT549 cells.

### 2.4. Cell Viability Analysis

The cells were plated in 96-well plates at a density of 5000 cells per well in a volume of 100 μL. The subsequent day, the cells were exposed to LV at concentrations of 0 and 10 μM (diluted in DMSO), and then incubated for 24 h in a controlled environment. A DMSO control group (0.1% DMSO without LV) was included to assess solvent effects. Cell viability was assessed using the Cell Counting Kit-8 (CCK-8, #B34304, Selleck, Houston, TX, USA) according to the manufacturer’s protocol. Three independent replicates were performed to ensure experimental reproducibility.

### 2.5. Soft Agar Assay

The cells were seeded in 6-well plates at a density of 8 × 10³ cells per well and incubated in Eagle’s basal medium at 37 °C for 2 weeks. Following incubation in the absence or presence of LV (0, 10 μM), colonies were visualized and counted using light microscopy.

### 2.6. Flow Cytometry Analysis

Apoptosis in TNBC cells was assessed using flow cytometry with an Annexin V-FITC/PI apoptosis kit (AP101, Multi Sciences, Hangzhou, China). Briefly, 1 × 10^6^ cells treated with various concentrations of LV (0, 5, 10, and 20 μM) were harvested, washed twice with cold PBS, and resuspended in 500 μL of 1 × binding buffer. The cells were then incubated with 5 μL of Annexin V-FITC and 10 μL of a PI viability staining solution for 5 min at room temperature in the dark. Finally, the cell suspension was analyzed by flow cytometry using BD FACSCanto™ II (BD Biosciences, San Jose, CA, USA).

### 2.7. Western Blot Assay

The cells were processed according to the experimental design. The cell precipitate was collected through digestion and centrifugation, washed with PBS, and then resuspended in a pre-cooled RIPA cell lysis buffer with a protease inhibitor (04693124001, Roche, Basel, Switzerland) to obtain the whole cell lysate (WCL). The protein concentration was measured using a BCA Protein Assay Kit (#23225, Thermo Fisher Scientific, Waltham, MA, USA), and the sample solution was prepared accordingly. Aliquots of the protein samples were separated by SDS-PAGE and transferred to a PVDF membrane. The membrane was blocked with 5% skimmed milk for 1 h and then incubated overnight at 4 °C with a primary antibody diluted in PBS. The following day, the primary antibody was removed, and the membrane was washed three times with TBST (5 min each). The membrane was then incubated with a secondary antibody conjugated with Horseradish Peroxidase (HRP). Target protein bands were detected using an Enhanced Chemiluminescence Reagent (ECL) (#34580, Thermo Fisher Scientific, Waltham, MA, USA).

### 2.8. Co-Immunoprecipitation

For the co-immunoprecipitation (Co-IP) assay, the cell lysate prepared from the cells treated with or without LV was incubated overnight at 4 °C with the indicated antibody and Protein A/G agarose beads. The beads were washed with pre-cooled PBS, then resuspended in 2 × SDS-PAGE loading buffer and boiled for 5 min to release the bound proteins. Subsequent experimental steps were performed as described above for Western blot analysis.

### 2.9. Subcellular Fraction Isolation

For experiments involving the extraction of mitochondrial and cytoplasmic proteins, both extracts were isolated from the cells treated with gradient concentrations of LV (0, 5, 10, and 20 μM) using the Mitochondria Isolation Kit for Cultured Cells (#89874, Thermo Fisher Scientific, Waltham, MA, USA). After treatment with LV for 24 h, the cells were collected and centrifuged at 850× *g* for 2 min at 4 °C to collect the cell pellets. Mitochondrial isolation reagent A was added to the cell pellets, mixed well, and incubated on ice for 2 min. Subsequently, mitochondrial isolation reagent B was added and incubated on ice for 5 min. Then, isolation reagent C was added to each tube, inverted to mix thoroughly, and centrifuged at 700× *g* for 10 min. After collecting the supernatant, a high-speed centrifugation was performed. The resulting supernatant represented the cytoplasmic fraction, while the precipitate was the mitochondrial fraction.

### 2.10. Immunofluorescence

After treatment of the cells with various concentrations of LV (0, 5, 10, and 20 μM), the medium was aspirated, and the cells were rinsed three times with PBS. The cells were then fixed in 4% paraformaldehyde for 10 min and permeabilized with 0.3% Triton X-100 for 20 min. After blocking, the cells were incubated overnight at 4 °C with the primary antibody, and the corresponding fluorescent secondary antibody was applied the next day. DAPI was utilized for nuclear staining, and a fluorescence microscope (DM4B, Leica, Wetzlar, Germany) was employed to observe the staining.

### 2.11. Quantitative Reverse-Transcription Polymerase Chain Reaction (qRT-PCR)

The total RNA was isolated from the treated cells using the TRIzol Reagent (Invitrogen, Carlsbad, CA, USA). The complementary DNA (cDNA) was synthesized through reverse transcription using the PrimeScriptTM RT Reagent Kit with gDNA Eraser (Takara, Tokyo, Japan). For quantitative reverse transcription PCR (qRT-PCR) analysis, SYBR Premix Ex Taq (Takara, Tokyo, Japan) was employed. The mix was amplified in a real-time PCR machine using cycles of pre-denaturation at 95 °C for 5 min, denaturation at 95 °C for 15 s, annealing at 60 °C for 30 s, and extension at 72 °C for 30 s, with fluorescence signals measured in real-time. Expression data were normalized to *GAPDH* as an internal control using the 2^−ΔΔCT^ method. All primers were chemically synthesized by Sangon Biotech (Shanghai, China).

### 2.12. Caspase 3 Activity Assay

Caspase 3 activity in TNBC cells treated with different concentrations of LV (0, 5, 10, and 20 μM) was assayed using the Caspase 3 Assay Kit (ab39401, Abcam, Cambridge, UK). Briefly, a reaction buffer and the DEVD-p-NA substrate were added to the cell lysates in the wells of the 96-well plate, followed by incubation for 60–120 min at 37 °C. The concentration of p-NA was analyzed using a microplate reader (Epoch, Bio-tek, Winooski, Vermont, USA) at 405 nm. The Caspase 3 activity was determined by comparing the absorbance of the samples with a standard curve.

### 2.13. Ubiquitination Analysis

After a 24 h LV (10 μM) treatment, the cells were collected and lysed, followed by sonication and boiling at 95 °C for 15 min. After heating, the lysates were centrifuged at 16,000× *g* for 10–15 min at 4 °C by microcentrifuge (Heraeus Fresco 21, Thermo, Waltham, MA, USA). The supernatant was collected, and the protein concentration was measured. A suitable volume of supernatant protein was combined with RIPA buffer containing 0.1% SDS to achieve a total volume of 750 µL, followed by the addition of the appropriate antibody and agarose beads. This mixture was incubated at 4 °C overnight. The following day, the agarose beads were washed three times with pre-cooled PBS, and the protein samples were prepared.

### 2.14. Cycloheximide Pulse-Chase Assay

The cells were pre-treated with LV (10 μM) for 48 h, followed by treatment with cycloheximide (CHX, 20 μg/mL) for 0, 2, 4, or 8 h. Whole cell lysates were then prepared at each time point for Western blot analysis to assess changes in the half-life of Survivin.

### 2.15. Xenograft Tumor Model

The impact of overexpressed USP14 on the tumorigenicity of TNBC cells in vivo was assessed using a mouse xenograft tumor model. All mice (6 weeks old, female) used in this study were purchased from the Department of Laboratory Animals of Central South University (Changsha, China). Nude mice were orthotopically injected with 5 × 10^6^ MDA-MB-231 cells (Vector or Flag-*USP14*-transfected) into the fourth mammary fat pad. The nude mice were randomly divided into four groups (5 mice/group) after injection: Vector + Vehicle, Vector + LV (10 mg/kg), Flag-*USP14* + Vehicle, and Flag-*USP14* + LV (10 mg/kg). Two to three weeks later, LV or normal saline (NS) was administered every two days via oral gavage. The mouse body weight and tumor volume were monitored and recorded every two days. Tumor growth was calculated using the formula (length × width^2^/2). Two weeks after LV treatment, the mice were euthanized, the tumor weights were recorded, and the tumor tissues were fixed in 4% formaldehyde for H&E staining and immunohistochemical analysis.

### 2.16. Immunohistochemistry

The tumor tissues were paraffin-embedded and cut into 3 μm-thick tissue sections. After deparaffinization and antigen retrieval, the sections were washed three times with distilled water and treated with 3% hydrogen peroxide at room temperature for 10 min to inhibit endogenous peroxidase activity. Next, the sections were blocked with goat serum for 45 min at room temperature and incubated overnight at 4 °C with the respective primary antibody. The following day, the sections were washed with PBS three times and incubated with the secondary antibody (PV-6000D, ZSGB, Beijing, China) for 45 min at room temperature. Finally, a DAB solution was added for color development, followed by counterstaining with hematoxylin.

### 2.17. Bioinformatics Analysis

Subtype-specific differential expression patterns of BIRC5 in BC were systematically interrogated through the GEPIA2 (http://gepia2.cancer-pku.cn, accessed on 13 April 2023) and cBioPortal (https://www.cbioportal.org/, accessed on 4 April 2023) databases. Furthermore, the prognostic relevance of BIRC5 expression in TNBC was assessed by Kaplan–Meier survival analysis using the Kaplan–Meier Plotter (https://kmplot.com, accessed on 17 October 2023), with statistical significance determined by the log-rank test.

### 2.18. Statistical Analysis

Quantitative data from at least three independent experiments were analyzed using the GraphPad Prism 8 software (version number: 8.0.1). Group comparisons were conducted using either Student’s *t*-test or one-way analysis of variance (ANOVA).

## 3. Results

### 3.1. LV Induces Apoptosis in TNBC Cells Through Activation of the Intrinsic Apoptotic Pathway

**Figure 1:**
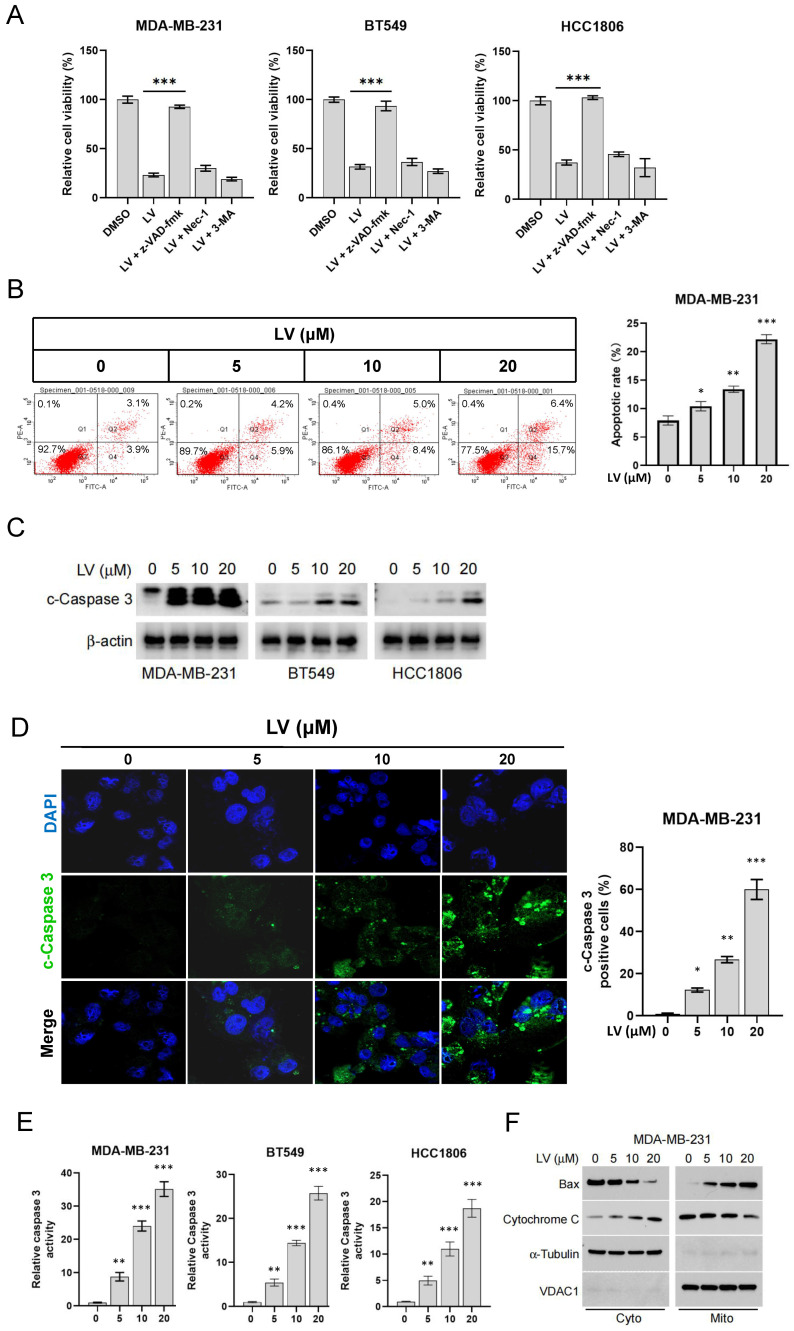
LV promotes apoptosis of TNBC cells. (**A**) MDA-MB-231, BT549, or HCC1806 cells were pre-treated for 4 h with the apoptosis inhibitor z-VAD-FMK, the necroptosis inhibitor Nec-1, or the autophagy inhibitor 3-MA, followed by treatment with LV (10 µM) for 24 h. Cell viability was measured using the CCK-8 assay. (**B**) Flow cytometry analysis of the effect of LV on the proportion of apoptotic cells in MDA-MB-231 cells. (**C**) Western blot analysis of the effect of different concentrations of LV on cleaved-Caspase 3 (c-Caspase 3) in MDA-MB-231, BT549, or HCC1806 cells. (**D**) Immunofluorescence examining the effect of LV on the proportion of c-Caspase 3-positive cells. (400×) (**E**) Caspase 3 activity in MDA-MB-231, BT549, or HCC1806 cells treated with different concentrations of LV, detected using a Caspase 3 activity assay kit. (**F**) After LV treatment, subcellular fractions of TNBC cells were isolated, and Western blot analyses were performed to detect the protein levels of Bax and cytochrome C in the mitochondrion and the cytoplasm. Data are presented as the mean ± SEM. * *p* < 0.05; ** *p* < 0.01; *** *p* < 0.001.

We have previously demonstrated that LV induces cell death in cultured TNBC cells and in a nude mouse model of the TNBC cell xenograft [24]. To further elucidate the molecular mechanism by which LV induces cell death in TNBC cells, we conducted intervention experiments using cell death inhibitors on LV-treated MDA-MB-231, BT549, and HCC1806 cells. These cells were pre-treated for 4 h with the apoptosis inhibitor z-VAD-FMK, the necroptosis inhibitor Nec-1, or the autophagy inhibitor 3-MA, followed by treatment with LV. We found that only the apoptosis inhibitor z-VAD-FMK significantly restored cell viability in MDA-MB-231, BT549, and HCC1806 cells post-LV treatment (Figure 1A), suggesting that apoptosis was the predominant form of cell death induced by LV in these cells. Flow cytometry revealed a significant increase in apoptotic cells upon LV treatment in MDA-MB-231 and BT549 cells (Figure 1B and Figure S1). Consistent with this, LV dose-dependently activated Caspase 3, as evidenced by Western blotting, immunofluorescence, and enzymatic activity assays (Figure 1C–E). Additionally, LV treatment significantly promoted the translocation of Bax from the cytoplasm into the mitochondrion and cytochrome C from the mitochondrion into the cytoplasm (Figure 1F), indicating that LV activates the intrinsic apoptotic pathway in TNBC cells.

### 3.2. LV Induces the Degradation of Survivin via the Ubiquitin–Proteasome Pathway in TNBC Cells

**Figure 2:**
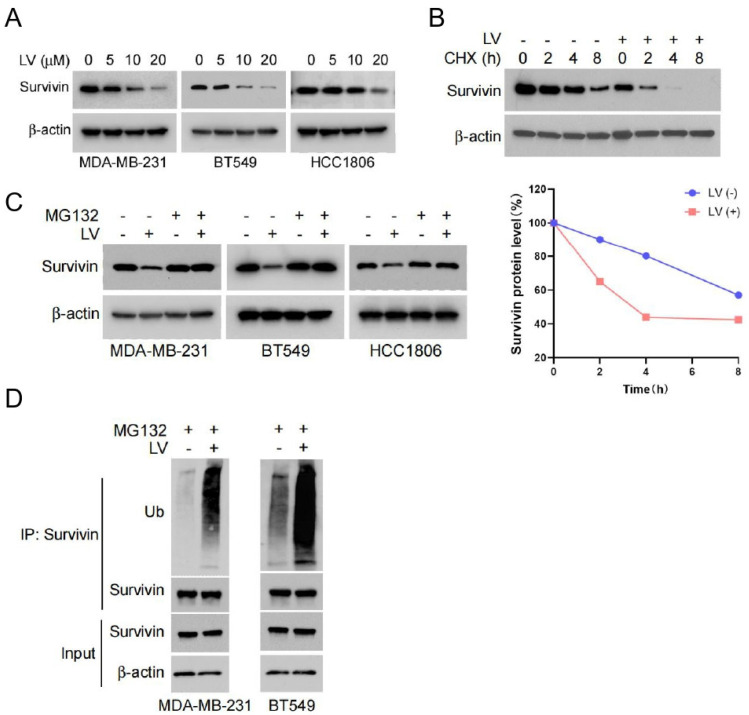
LV shortens the half-life of Survivin and influences its degradation via the ubiquitin–proteasome pathway. (**A**) The protein levels of Survivin detected by Western blot analysis in MDA-MB-231, BT549, or HCC1806 cells treated with different concentrations of LV. (**B**) The protein levels of Survivin in BT549 cells treated with or without LV after CHX treatment for 0, 2, 4, or 8 h, detected by Western blot analysis. (**C**) The effect of LV on the protein level of Survivin in MDA-MB-231, BT549, or HCC1806 cells in the presence or absence of MG132, detected by Western blot analysis. (**D**) The ubiquitination level of Survivin in MDA-MB-231 or BT549 cells upon treatment with or without LV (10 µM), measured by Co-IP assay.

In order to reveal the molecular mechanism by which LV induces apoptosis in TNBC cells, we assessed the expression of apoptosis-related proteins in LV-treated TNBC and non-TNBC cells by Western blotting. One of the predominant findings was that LV dose-dependently downregulated the protein level of Survivin in TNBC MDA-MB-231, BT549, and HCC1806 cells (Figure 2A). However, qRT-PCR revealed that LV did not affect the mRNA expression of *Survivin* (Figure S2), suggesting that the regulation of Survivin by LV may occur through protein degradation. We next conducted a CHX pulse-chase experiment to reveal the effect of LV on Survivin protein stability. As expected, LV treatment led to a more pronounced downregulation of Survivin in BT549 cells (Figure 2B). Moreover, the proteasome inhibitor MG132 effectively reversed the reduction in Survivin levels induced by LV (Figure 2C). The ubiquitination analysis further revealed that LV significantly promoted ubiquitination in both MDA-MB-231 and BT549 cells (Figure 2D). Functionally, the knockdown of *Survivin* using lentivirus-mediated gene silencing significantly inhibited the ability of TNBC cells to form colonies in soft agar (Figure S3A,B). These findings suggest that LV induces apoptosis in TNBC cells by regulating the stability of Survivin through the ubiquitin–proteasome pathway.

### 3.3. LV Destabilizes Survivin by Disrupting the Interaction Between USP14 and Survivin in TNBC Cells

**Figure 3:**
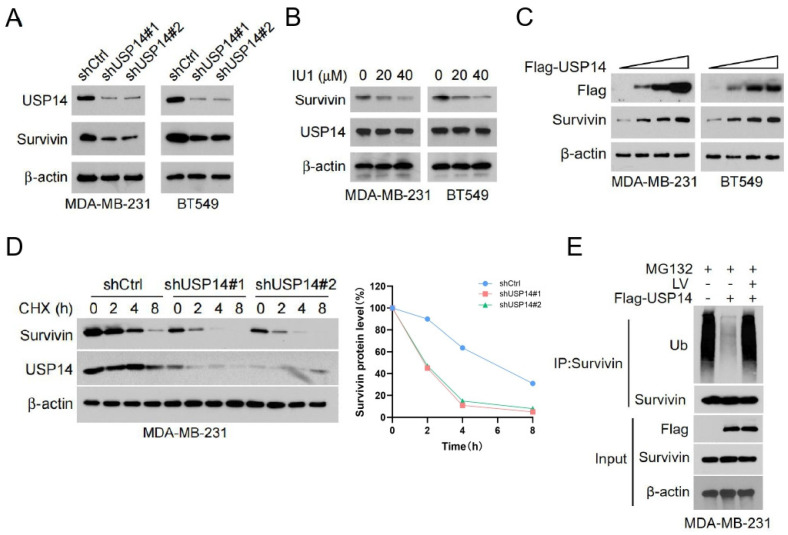
USP14 is responsible for mediating LV’s regulation of Survivin ubiquitination and stability. (**A**) Western blot analysis of USP14 and Survivin in MDA-MB-231 or BT549 cells with or without USP14 silencing. (**B**) The protein levels of Survivin in MDA-MB-231 or BT549 cells treated with the USP14 inhibitor IU1 (0, 10, or 20 µM). (**C**) The protein levels of Survivin in MDA-MB-231 or BT549 cells with USP14 overexpression, detected by Western blot analysis. (**D**) The protein levels of Survivin and USP14 in MDA-MB-231 cells with or without USP14 silencing, detected by Western blot analysis after CHX treatment for 0, 2, 4, or 8 h. (**E**) The ubiquitination level of Survivin upon overexpression of USP14 and treatment with LV, detected by Co-IP assay.

We further investigated the deubiquitinating enzymes that may regulate the stability of Survivin. TIMER2.0 (http://timer.cistrome.org/, accessed on 16 July 2023) is an online platform for tumor immune analysis, which can provide various data analyses of tumor immune-related functions [27]. Using TIMER2.0, we conducted a correlation analysis to examine the relationship between *Survivin* and genes belonging to the family of ubiquitin-specific proteases (USPs) in TNBC. Among all the USPs analyzed, USP14 was found to be the most potent deubiquitinase in protecting the stability of Survivin. To corroborate this notion, we performed experiments on TNBC cells with *USP14* gene silencing. Notably, the gene silencing of *USP14* resulted in a significantly decreased level of Survivin in MDA-MB-231 and BT549 cells (Figure 3A). Furthermore, treatment with the USP14 inhibitor IU1 [25] resulted in a dose-dependent reduction in the protein levels of Survivin in both cell lines (Figure 3B). In contrast, the overexpression of *USP14* led to a dose-dependent increase in Survivin protein expression in MDA-MB-231 and BT549 cells (Figure 3C). CHX pulse-chase experiments demonstrated that the silencing of *USP14* significantly reduced the half-life of Survivin protein (Figure 3D), indicating that *USP14* plays a crucial role in maintaining its stability. Additionally, the ubiquitination assay revealed that the overexpression of USP14 markedly inhibited the ubiquitination levels of Survivin. Moreover, LV treatment reversed the inhibitory effect of USP14 on the ubiquitination levels of Survivin (Figure 3E).

**Figure 4:**
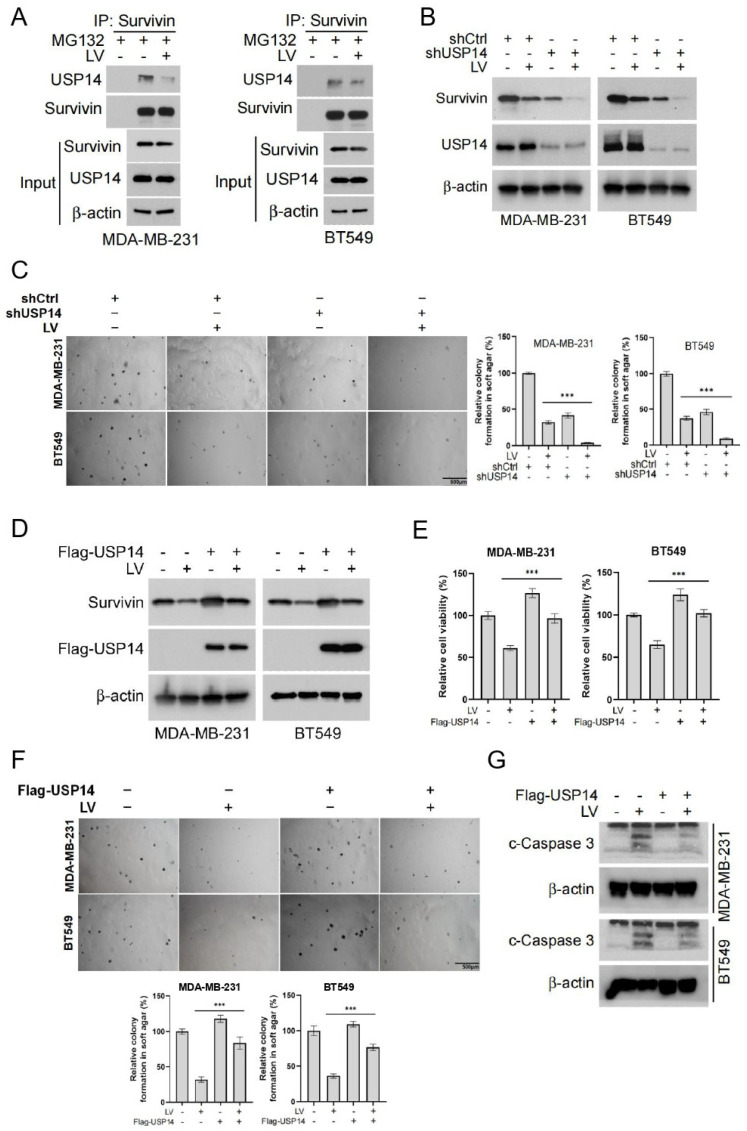
*USP14* stabilizes the Survivin protein and plays a critical role in regulating the sensitivity of TNBC cells to LV. (**A**) The interaction between USP14 and Survivin upon treatment with MG132 and LV, detected by Co-IP assay. (**B**) The protein level of Survivin in MDA-MB-231 or BT549 cells with *USP14* gene silencing, with or without LV treatment detected by Western blot analysis. (**C**) The effect of LV on colony formation after *USP14* silencing in MDA-MB-231 or BT549 cells, analyzed by soft agar colony formation assay. (**D**) The effect of LV on Survivin in MDA-MB-231 or BT549 cells with *USP14* overexpression, detected by Western blot analysis. The effect of LV on cell viability (**E**) and colony formation (**F**) after *USP14* overexpression in MDA-MB-231 or BT549 cells, analyzed by CCK8 and soft agar colony formation assay, respectively. (**G**) The level of c-Caspase 3 in MDA-MB-231 or BT549 cells with or without *USP14* overexpression, treated with or without LV, and detected by Western blot analysis. Data are shown as the mean ± SEM. *** *p* < 0.001.

To further investigate the regulation of Survivin by *USP14*, we examined the interaction between USP14 and Survivin using a Co-IP assay. We found that USP14 and Survivin formed a complex, and this binding was reduced following LV treatment (Figure 4A). Next, we established a stable cell line with *USP14* gene silencing, which demonstrated that silencing *USP14* enhanced the downregulation of Survivin by LV (Figure 4B). Soft agar assay and CCK8 analysis revealed that *USP14* silencing increased the sensitivity of MDA-MB-231 and BT549 cells to LV, resulting in a greater inhibitory effect on both colony formation and cell viability (Figure 4C and Figure S4). Moreover, the overexpression of *USP14* in MDA-MB-231 and BT549 cells blocked the downregulation of Survivin that is induced by LV (Figure 4D). Consequently, *USP14* overexpression resulted in a reduction in cell viability and colony formation normally observed with LV treatment, suggesting that the overexpression of *USP14* significantly reduced the sensitivity of these cells to LV (Figure 4E,F). Western blot analysis further showed that overexpression of *USP14* inhibited LV-induced apoptosis in MDA-MB-231 and BT549 cells, as evidenced by a markedly decreased level of cleaved-Caspase 3 (Figure 4G). These findings indicate that *USP14* stabilizes the Survivin protein and plays a crucial role in regulating the sensitivity of TNBC cells to LV.

### 3.4. The Overexpression of USP14 Abrogates the Anti-Tumor Activity of LV In Vivo

**Figure 5:**
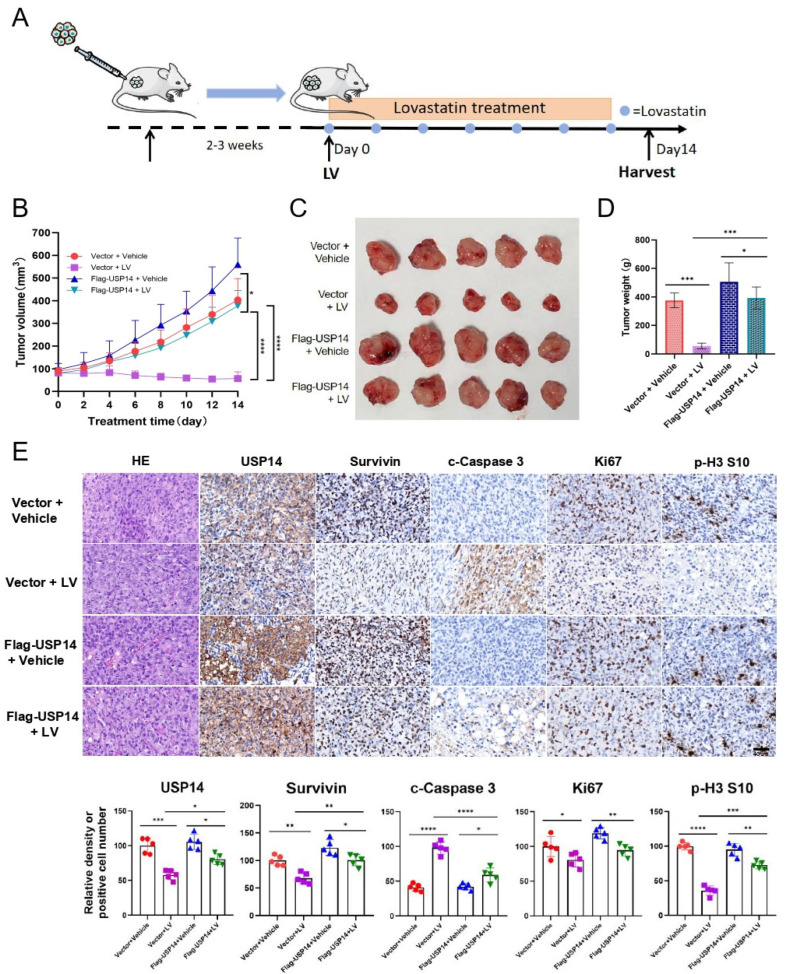
LV inhibits Survivin in vivo, and the overexpression of *USP14* impairs the anti-tumor effects of LV. (**A**) A diagram illustrating the animal experimental design. (**B**) The change of tumor volume over time from different groups. (**C**) A photograph of tumors isolated from the nude mice. (**D**) The tumor weights of the mice from different groups. (*n* = 5/group). (**E**) The protein levels of USP14, Survivin, c-Caspase 3, Ki67, or p-H3 in the four groups detected by immunohistochemistry (×400 magnification). The corresponding quantifications are illustrated at the bottom. Data are shown as the mean ± SEM. * *p* < 0.05; ** *p* < 0.01; *** *p* < 0.001; **** *p* < 0.0001.

To investigate the effect of *USP14* on LV’s regulation of Survivin protein stability in vivo, we used a mouse xenograft tumor model established with MDA-MB-231 cells (Figure 5A). The overall in vivo toxicity of LV was found to be minimal, as determined by no obvious change in the mouse body weight (Figure S5A) and no significant toxicity to organs such as the heart, liver, spleen, lungs, and kidneys (Figure S5B). We found that while LV significantly inhibited the tumor growth, tumor volume, and tumor weight of MDA-MB-231-derived xenograft tumors in vivo, the overexpression of *USP14* significantly reversed these inhibitory effects of LV (Figure 5B–D). Immunohistochemistry indicated that LV significantly decreased the protein level of Survivin in tumor tissues. However, this inhibitory effect was compromised in MDA-MB-231 tumors with *USP14* overexpression. Additionally, LV treatment decreased the population of Ki67 and p-H3 S10-positive cells and increased the number of cleaved-Caspase 3-positive cells. As expected, the overexpression of *USP14* reversed the effects of LV on the expression of these proteins (Figure 5E). These findings suggest that LV can decrease the Survivin protein level in vivo and that the overexpression of *USP14* may stabilize Survivin and impair the anti-tumor effects of LV in TNBC.

### 3.5. Higher Levels of Survivin Predict Poorer Prognosis in TNBC Patients

**Figure 6:**
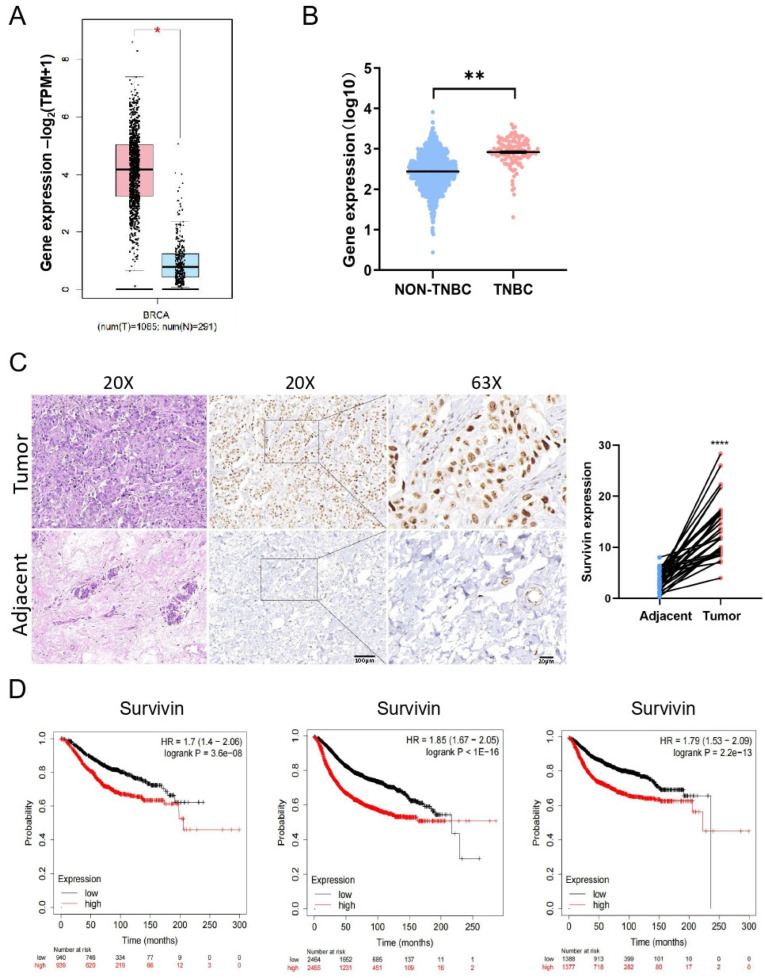
Survivin is expressed at higher levels in BC and correlates with poor prognosis. (**A**,**B**) The mRNA levels of *Survivin* evaluated between TNBC tissues and normal breast tissues (**A**) from GEPIA2 or between TNBC and non-TNBC tissues (**B**) using the data from cBioPortal. (**C**) The protein levels of Survivin in TNBC tissues and adjacent normal tissues assessed using immunohistochemistry (×200 and ×630 magnification). (**D**) Kaplan–Meier survival analysis of the prognosis between TNBC patients with high- and low-expression of Survivin. Data are shown as the mean ± SEM. * *p* < 0.05; ** *p* < 0.01; **** *p* < 0.0001.

To investigate the clinical significance of Survivin in TNBC patients, we first analyzed the mRNA levels of *Survivin* in normal breast tissues and cancer from GEPIA2. We found that the mRNA expression of *Survivin* in TNBC tissues was significantly higher in BC tissues compared with normal breast tissues (Figure 6A) and was significantly higher in TNBC than in non-TNBC breast cancer tissues (Figure 6B). Immunohistochemistry showed that the protein level of Survivin was significantly increased in TNBC tissues compared with adjacent non-cancerous tissues (Figure 6C). Furthermore, the higher expression of Survivin in TNBC patient tissues was associated with poorer prognosis for overall survival (OS), relapse-free survival (RFS), and distant metastasis-free survival (DMFS) in TNBC patients (Figure 6D). These results suggest that higher levels of the Survivin gene or protein are indicative of poorer prognosis in TNBC patients.

## 4. Discussion

TNBC is one of the most aggressive subtypes of BC, characterized by high malignancy and poor prognosis, which imposes significant physical, emotional, and socio-economic burdens on patients, their families, and society at large [3]. In the present study, we observed that Survivin exhibits high expression levels in patient-derived tumor tissues. It has been demonstrated that high levels of Survivin expression are strongly associated with poor prognosis and tumor metastasis in cancer patients. For instance, esophageal squamous cell carcinoma (ESCC) frequently develops lung metastases, and it has been suggested that the depletion of the *Survivin* gene specifically reduces *Trp53R172H* (a hotspot mutation in *TP53*)-driven lung metastases, and that high levels of *Survivin* expression are associated with increased metastasis in several gastrointestinal cancers [28,29]. Furthermore, a previous report also showed that elevated *Survivin* levels may serve as an independent biomarker of poor prognosis in patients with oral squamous carcinoma after surgical resection [30]. As a core member of the IAP family, *Survivin* not only directly regulates apoptosis but also promotes tumor progression and metastasis by engaging in signaling pathways such as NF-κB and ERK1/2. Activated by TNF-α, NF-κB promotes *Survivin* expression, while *Survivin* reinforces NF-κB activity via a positive feedback loop by relieving the IκB-mediated inhibition of NF-κB nuclear translocation. In addition, the activation of the ERK signaling pathway is also associated with the upregulation of *Survivin* expression. These findings underscore the potential of *Survivin* as a therapeutic target in cancer treatment. However, the clinical development of *Survivin*-targeted therapies is challenged by the lack of the protein’s enzymatic activity and active sites suitable for small-molecule binding or inhibition [31,32].

The turnover of the Survivin protein is intricately regulated by ubiquitinases and deubiquitinases [33,34,35,36,37]. Although the predominant ubiquitin chain topology (e.g., K48 vs. K63 linkages) involved in *USP14*-mediated *Survivin* stabilization remains to be fully elucidated, *USP14* has been discovered to be highly expressed across various cancer types and plays a critical role in regulating protein stability and degradation within several important signaling pathways, including Survivin in other cancers [25,26]. The role of *USP14* in regulating the sensitivity of retinoblastoma (RB) cells to cisplatin (DDP) and its potential mechanism were explored [38]. Their results showed that the level of *Survivin* was significantly reduced in RB cell lines with the deletion of *USP14*, indicating that *USP14* plays an important role in regulating the protein stability of *Survivin*. Notably, while our mechanistic studies focused on the direct *USP14–Survivin* axis, potential upstream regulators such as kinase cascades or epigenetic modifiers that modulate this interaction in TNBC merit further exploration.

**Figure 7:**
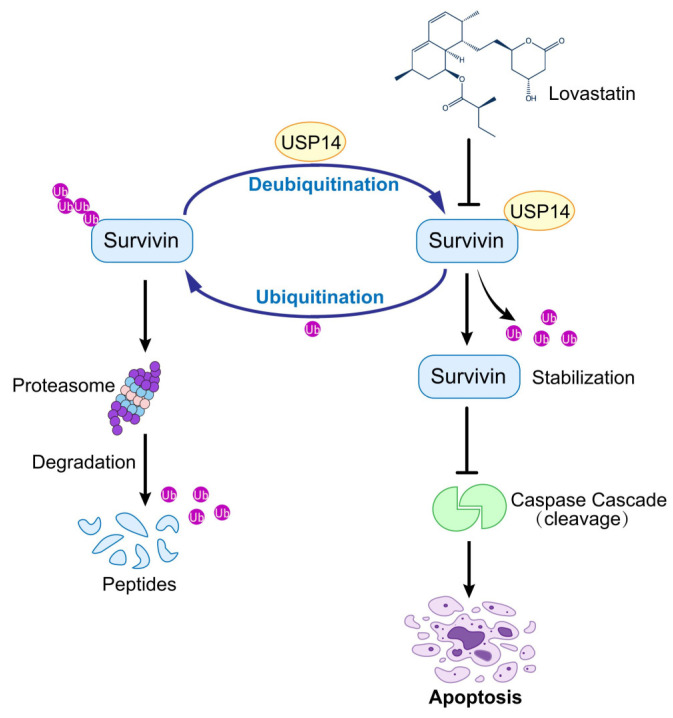
LV exhibits anti-tumor activity against TNBC by targeting the USP14–Survivin axis. LV disrupts the interaction between USP14 and Survivin in TNBC, leading to the inhibition of USP14’s deubiquitinating activity on Survivin and consequently enhancing the ubiquitination and degradation of Survivin.

In our study, we showed that the inhibition of *USP14* decreased the protein level of Survivin and shortened its half-life in TNBC cell lines. Conversely, the overexpression of *USP14* enhanced the protein level of Survivin, decreased its ubiquitination, and reversed the inhibitory effects of LV on TNBC. In vivo experiments further demonstrated that *USP14* overexpression significantly attenuated the inhibitory effect of LV on TNBC xenograft tumors, but did not significantly alter Ki67 or p-H3 S10 (proliferation markers) beyond reversing LV’s effects. Intriguingly, the preclinical evaluation of combinatorial approaches targeting both *USP14* (e.g., IU1) and statins may enhance therapeutic efficacy, though this hypothesis requires systematic investigation in appropriate animal models. Further mechanistic studies showed that LV promoted ubiquitination and the degradation of Survivin by interfering with the interaction between USP14 and Survivin, rather than regulating the protein levels of USP14. Our preliminary data suggest that *USP14* silencing may broadly enhance TNBC cell sensitivity to diverse apoptosis-inducing agents. The disrupted interaction between USP14 and Survivin led to the increased ubiquitination of Survivin, followed by increased proteasome-mediated degradation (Figure 7).

While our current findings demonstrate Survivin degradation as a key pharmacodynamic event, its potential utility as a predictive biomarker for statin-based therapies in TNBC patients requires further validation through prospective clinical studies. The development of dual-targeting strategies against both *USP14* and *Survivin* could theoretically mitigate drug resistance mechanisms, though the feasibility and safety profile of such approaches need rigorous preclinical evaluation.

## 5. Conclusions

In summary, our study demonstrated that LV disrupts the interaction between USP14 and Survivin, which, in turn, promotes the ubiquitination and degradation of Survivin and induces apoptosis in TNBC cells. Our findings elucidate a critical role for *USP14* in the regulation of Survivin in TNBC cells. Therefore, the *USP14–Survivin* axis may represent a promising target for the treatment of TNBC, which deserves further investigation before its recognition as a clinically actionable scheme.

## Data Availability

The datasets used and/or analyzed during the current study are available from the corresponding author upon reasonable request.

## Supplementary Materials

The following supporting information can be downloaded at https://www.mdpi.com/article/10.3390/cells14110816/s1. Supplementary Figure S1. LV notably increases the proportion of apoptotic cells in TNBC cell. LV significantly increased the proportion of apoptotic cells in the BT549 cell line in a dose-dependent manner. LV, lovastatin; n.s., not significant; * *p* < 0.05, ** *p* < 0.01. Supplementary Figure S2. LV does not affect the mRNA expression of *Survivin*. Quantitative RT-PCR (qRT-PCR) showing that LV did not affect the mRNA expression of *Survivin* in MDA-MB-231 and BT549 cells. LV, lovastatin; ns, not significant. Supplementary Figure S3. Gene silencing of *Survivin* inhibits colony formation in TNBC cells. (A) Stable cell lines with *Survivin* knockdown were established using lentivirus-mediated gene silencing. The knockdown efficiency of *Survivin* in MDA-MB-231, BT549, and HCC1806 cells was verified by Western blotting. (B) The colony formation ability was measured by soft agar colony formation assay in *Survivin* knockdown cells or control (Ctrl) cells. *** *p* < 0.001. Supplementary Figure S4. *USP14* gene silencing potentiates the inhibitory effect of LV on cell viability in TNBC cell lines. The effect of LV on cell viability in MDA-MB-231 and BT549 cells with or without *USP14* gene silencing was analyzed by CCK8 assay. LV, lovastatin; *** *p* < 0.001. Supplementary Figure S5. Toxicity evaluation of LV treatment in mice. (A) Effect of LV treatment on the weight of nude mice. (B) Safety evaluation of organ tissues by microscopic pathological analysis (400×). H&E staining was performed on tissue sections for pathological assessment. LV, lovastatin.
