# Supplementary material for: Lovastatin Targets the USP14–Survivin Axis to Suppress Triple-Negative Breast Cancer via Ubiquitin-Mediated Proteasomal Degradation
Source: Cells. 2025 May 31;14(11):816. doi: 10.3390/cells14110816 (PMC12154129; doi:10.3390/cells14110816)
Supplement: Supplementary file 1 [file cells-14-00816-s001.zip › cells-3624888-supplementary.pdf]

Supplementary Material

1 Supplementary Figures

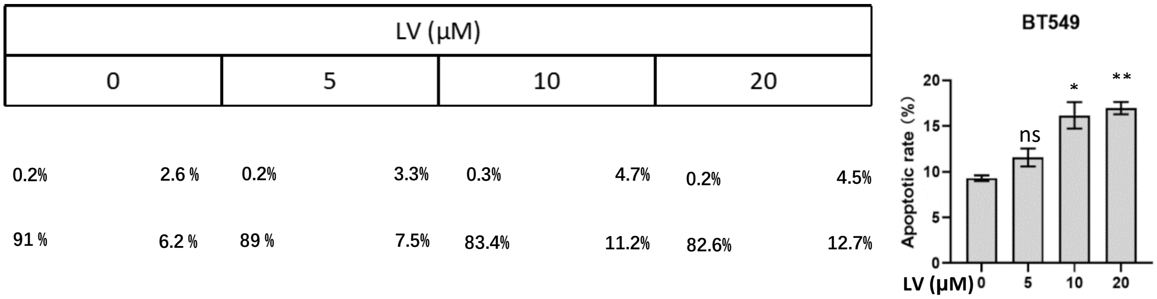

Supplementary Figure S1. LV notably increases the proportion of apoptotic cells in TNBC cell.

LV significantly increased the proportion of apoptotic cells in the BT549 cell line in a dose-dependent manner. LV, lovastatin; n.s., not significant; *P < 0.05, **P < 0.01.

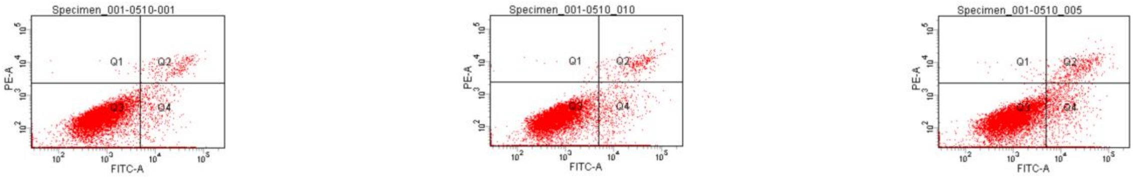

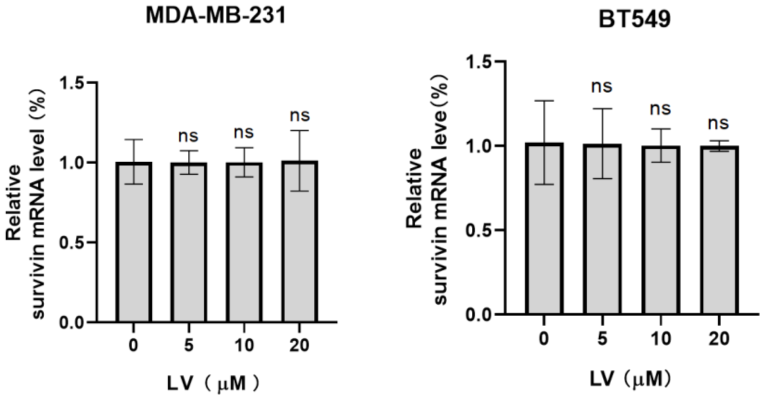

Supplementary Figure S2. LV does not affect the mRNA expression of *Survivin*. Quantitative RT-PCR (qRT-PCR) showing that LV did not affect the mRNA expression of S*urvivin* in MDA-MB-231 and BT549 cells. LV, lovastatin; ns, not significant.

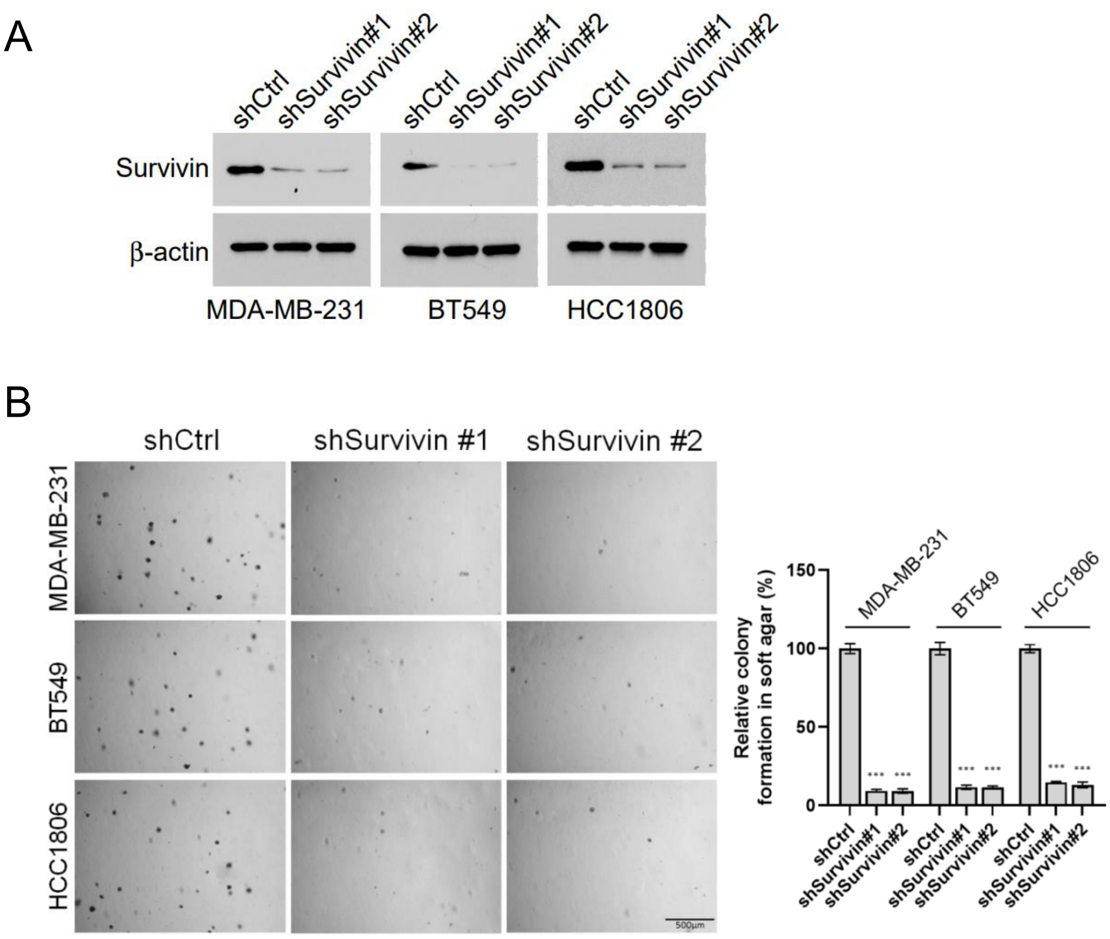

Supplementary Figure S3. Gene silencing of *Survivin* inhibits colony formation in TNBC cells. (A) Stable cell lines with *Survivin* knockdown were established using lentivirus-mediated gene silencing. The knockdown efficiency of *Survivin* in MDA-MB-231, BT549, and HCC1806 cells was verified by Western blotting. (B) The colony formation ability was measured by soft agar colony formation assay in *Survivin* knockdown cells or control (Ctrl) cells. ***$P < 0.001$.

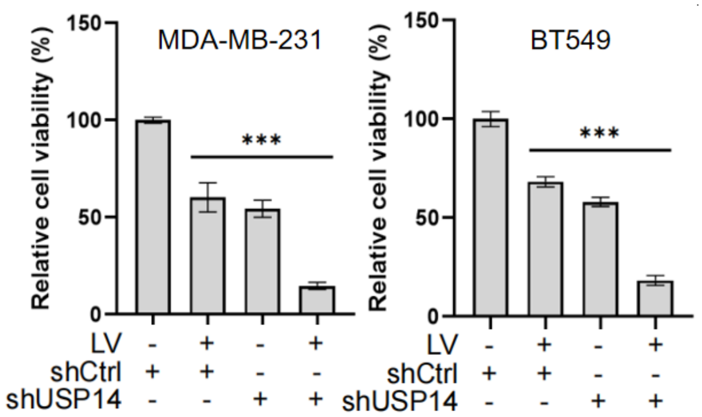

Supplementary Figure S4. *USP14* gene silencing potentiates the inhibitory effect of LV on cell viability in TNBC cell lines. The effect of LV on cell viability in MDA-MB-231 and BT549 cells with or without *USP14* gene silencing was analyzed by CCK8 assay. LV, lovastatin; ***$P < 0.001$.

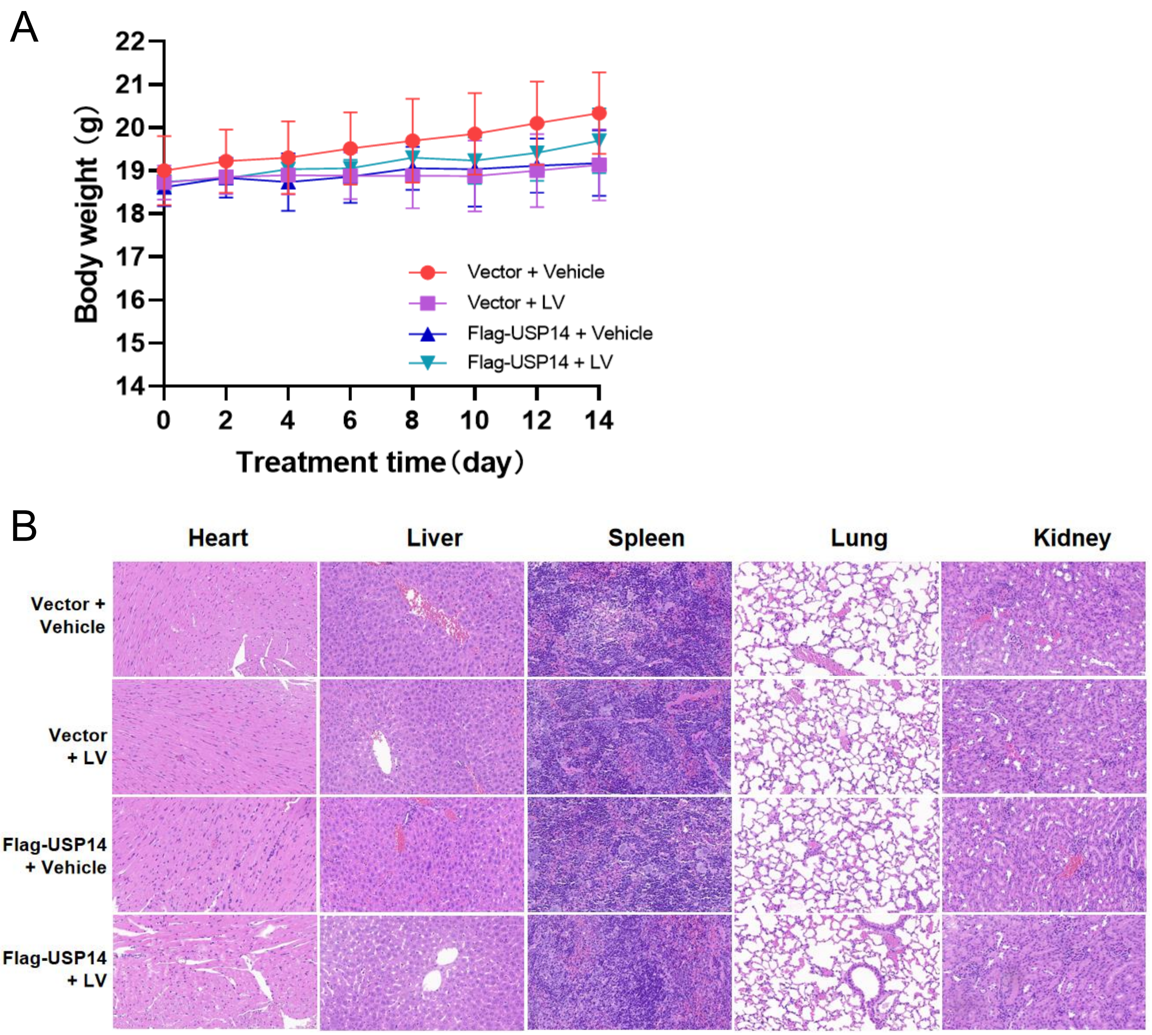

Supplementary Figure S5. Toxicity evaluation of LV treatment in mice. (A) Effect of LV treatment on the weight of nude mice. (B) Safety evaluation of organ tissues by microscopic pathological analysis (400×). H&E staining was performed on tissue sections for pathological assessment. LV, lovastatin.
